# Extracellular Matrix in Aging Aorta

**DOI:** 10.3389/fcell.2022.822561

**Published:** 2022-02-21

**Authors:** Akiko Mammoto, Kienna Matus, Tadanori Mammoto

**Affiliations:** ^1^ Department of Pediatrics, Milwaukee, WI, United States; ^2^ Department of Cell Biology, Neurobiology and Anatomy, Milwaukee, WI, United States; ^3^ Department of Pharmacology and Toxicology, Medical College of Wisconsin, Milwaukee, WI, United States

**Keywords:** extracellular matrix, aging, aorta, stiffness, elastin, collagen

## Abstract

The aging population is booming all over the world and arterial aging causes various age-associated pathologies such as cardiovascular diseases (CVDs). The aorta is the largest elastic artery, and transforms pulsatile flow generated by the left ventricle into steady flow to maintain circulation in distal tissues and organs. Age-associated structural and functional changes in the aortic wall such as dilation, tortuousness, stiffening and losing elasticity hamper stable peripheral circulation, lead to tissue and organ dysfunctions in aged people. The extracellular matrix (ECM) is a three-dimensional network of macromolecules produced by resident cells. The composition and organization of key ECM components determine the structure-function relationships of the aorta and therefore maintaining their homeostasis is critical for a healthy performance. Age-associated remodeling of the ECM structural components, including fragmentation of elastic fibers and excessive deposition and crosslinking of collagens, is a hallmark of aging and leads to functional stiffening of the aorta. In this mini review, we discuss age-associated alterations of the ECM in the aortic wall and shed light on how understanding the mechanisms of aortic aging can lead to the development of efficient strategy for aortic pathologies and CVDs.

## Introduction

The aging population is booming worldwide. At present, those aged over 65 years old constitute 16.5% of the population in the United States (US), and this number is expected to rise to 22% by 2050 (https://www.census.gov/content/dam/Census/library/publications/2020/demo/p25-1146.pdf). Aging is associated with structural and functional alterations in tissues and organs that over time, lead to various age-associated pathologies (Donato et al., 2018; Franceschi et al., 2018). The arteries transport circulating cells, oxygen and nutrients to local tissues and organs. The structure and function of the arteries are significantly altered during aging, which makes aging a major risk factor for cardiovascular diseases (CVDs) (Heidenreich et al., 2011; North and Sinclair, 2012; Paneni et al., 2017). Arterial aging is characterized by increased stiffness, reduced elasticity, impaired distensibility, endothelial dysfunction, and deregulated vascular tone (Paneni et al., 2015; Sun, 2015). To develop more efficient treatment to slow down arterial aging and prevent age-associated pathologies, it is necessary to comprehensively understand the structural and functional alterations in the aging arteries.

The extracellular matrix (ECM) is a three-dimensional network of macromolecules that determines morphological and physical properties of the tissues and organs (Frantz et al., 2010; Theocharis et al., 2016). Homeostasis of the ECM network is critical for maintaining tissue structure-functional relationships, and aberrant ECM remodeling contributes to various pathological conditions in the aging population (Birch, 2018; Theocharis et al., 2019). ECM proteins such as elastin, collagens, and soluble proteoglycans are the major components of the arterial wall (Jana et al., 2019). In large arteries, the ECM provides a structural framework, which not only withstands a wide range of tensile stresses, but also preserves its shape and functionality (Wagenseil and Mecham, 2009; Beenakker et al., 2012). In addition to structure, the ECM provides signaling cues that regulate proliferation and differentiation of resident cells [vascular smooth muscle cells (VSMCs), endothelial cells (ECs)] in the arterial wall (Davis and Senger, 2008; Nakayama et al., 2014).

The aorta is the largest elastic artery and the ascending region is anatomically positioned at the top of the left ventricle, which then arches (aortic arch) and branches into smaller arteries (right and left carotid and subclavian), before descending into the abdominal area. Elasticity of the aorta facilitates distension and recoil of the aortic wall during cardiac cycles to transform pulsatile flow generated by the left ventricle into steady flow to maintain local circulation in tissues and organs (Belz, 1995; Westerhof et al., 2009). The ECM composition and its three-dimensional structure determine morphology, mechanical property and functionality of the aorta (Wagenseil and Mecham, 2009). For example, elastic fibers and associated microfibrils and proteoglycans in the media (Halper and Kjaer, 2014; Hedtke et al., 2019; Halabi and Kozel, 2020) allow the aorta to expand and recoil during cardiac cycles (Cocciolone et al., 2018). On the other hand, fibrillar collagens (predominantly collagen I and III) in the media and adventitia are responsible for the tensile strength of the aortic wall to withstand the axial pressure created by the beating left ventricle (Wagenseil and Mecham, 2009). Along aging, elastic fibers in the aortic wall are progressively fragmented and lose their original elastic properties due to the life-long mechanical loads and low-grade chronic inflammation (Antonicelli et al., 2007; Duca et al., 2016; Heinz, 2021). Morphologically, the aortic aging is characterized by dilation, tortuousness and wall thickening in the intima and media layers (Gariepy et al., 1998; Lakatta and Levy, 2003), predominantly resulting from structural remodeling of the ECM and deregulated behaviors of VSMCs (Collins et al., 2014b). Fragmentation of the elastic fibers, aberrant collagen deposition and excess crosslinking of these ECM molecules lead to loss of elasticity and stiffening of the aortic wall (Qiu et al., 2007; Kohn et al., 2015; Duca et al., 2016). Aortic stiffness is known to parallel aortic aging and has been regarded as an independent predictor of morbidity and mortality of CVDs in the aging population (Mceniery et al., 2007; Wallace et al., 2007; Mitchell et al., 2010); aortic stiffening precedes clinical hypertension and is considered as the earliest predictor of hypertension (Liao et al., 1999; Dernellis and Panaretou, 2005; Peralta et al., 2010; Sun, 2015). In this mini review, we discuss the role of aberrant ECM remodeling in aging aorta and the underlying mechanism.

## Anatomy of Aorta

### Intima

**FIGURE 1:**
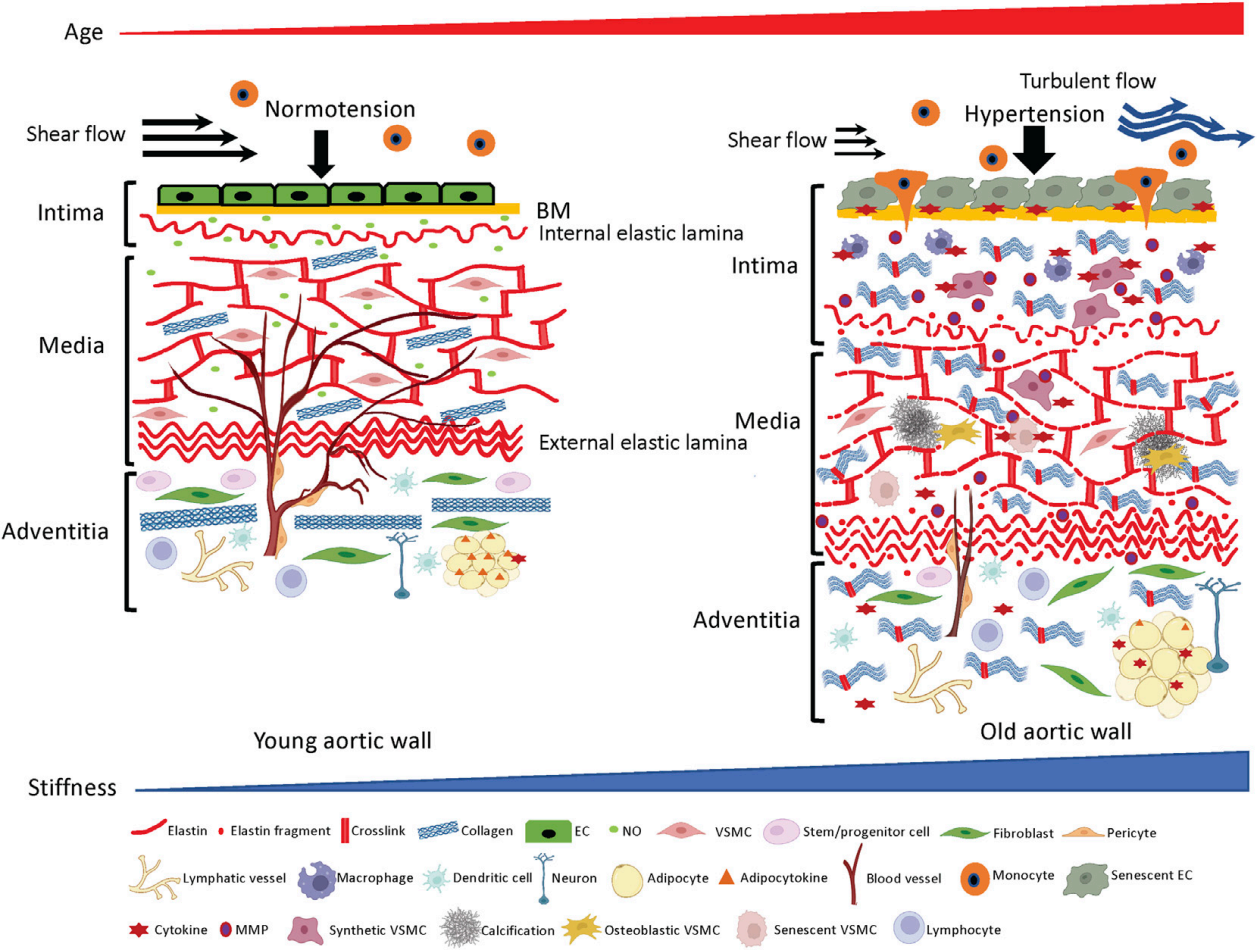
ECM structures and cellular components in the aortic wall. Young aortic wall (*left*): In intima, endothelial cells (ECs) maintain homeostasis of the wall by forming seamless barrier structure over the basement membrane (BM) and producing vasoprotective factors such as nitric oxide (NO). In media, key ECM molecules (e.g., elastin, collagen) and vascular smooth muscle cells (VSMCs) create the contractile units to maintain vascular tone and compliance. In adventitia, collagen fibers support the aortic wall to prevent overexpansion, and various adventitial cellular and non-cellular ECM components maintain homeostasis of the aortic wall. Aged aortic wall (*right*): Aging induces senescence of the ECs, which leads to chronic low-grade inflammation and subsequent aberrant ECM remodeling (fragmentation of elastin, excess deposition of collagen and their crosslinking) in the intima and media. Adventitial fibroblasts directly or indirectly stiffen the aortic wall by depositing excessive collagens.

The intima is the innermost layer of the aortic wall composed of a single layer of ECs, basement membrane (BM) and subendothelial space (Figure 1, left panel). ECs form a seamless monolayer over the BM, a thin pliable ECM membrane mainly consisting of laminin, collagen IV, fibronectin, perlecan, and heparan sulfate proteoglycans (Hallmann et al., 2005; Yurchenco, 2011). The BM plays crucial roles in the signaling events that regulate migration, proliferation, survival and barrier functions of resident vascular ECs (Davis and Senger, 2005). The vascular endothelium regulates homeostasis of the vessel by producing various vasoprotective factors such as nitric oxide (NO) to maintain vascular tone and blood flow (Sandoo et al., 2010; Kruger-Genge et al., 2019). The single layer of internal elastic lamina not only physically supports ECs and the BM, but also acts as a barrier structure between intima and media to prevent infiltration of circulating factors and media-derived cells that may trigger pathogenesis.

### Media

**FIGURE 2:**
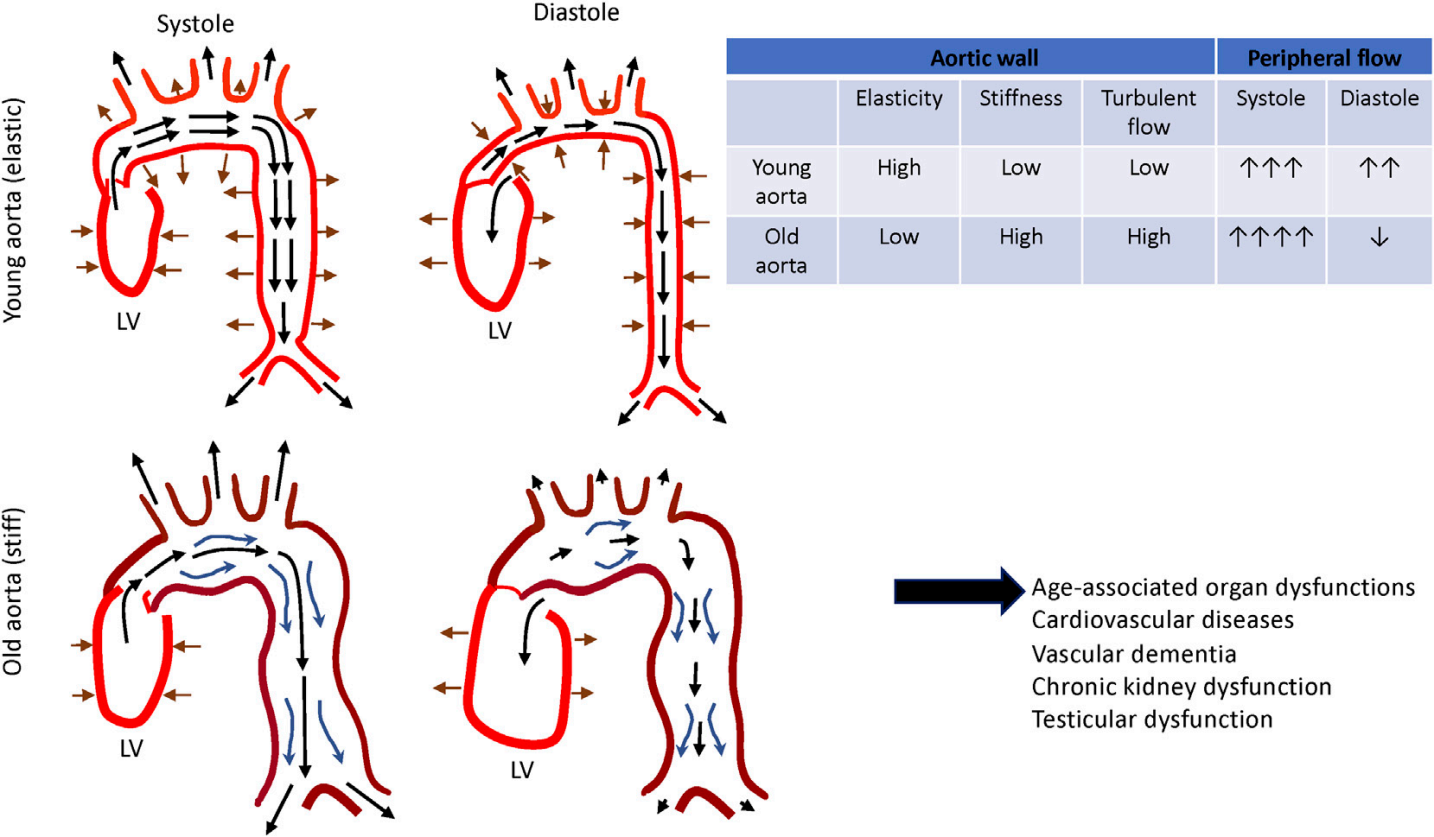
Changes in aortic functions during aging. Aortic wall stores energy generated by the left ventricle during systole and release the energy during diastole by returning to its initial dimension. This facilitates smooth and constant circulation in the peripheral tissues and organs (*top*). Aged aorta becomes dilated and tortuous. Together with these morphological changes, thickening and stiffening of the aging aortic wall disturb the local circulation, which leads to the tissue and organ dysfunction (*bottom*).

The media layer of the vessel is predominantly composed of the lamellar units of elastic fibers, collagen fibers and VSMCs (Clark and Glagov, 1985; Tsamis et al., 2013) (Figure 1, left panel) and determines the elastic properties of the aortic wall (Taghizadeh et al., 2015). Elastic fibers and VSMCs create a highly organized media layer within the vessel wall. VSMCs synthesize tropoelastin, which is a water-soluble precursor to elastin (Ozsvar et al., 2021). Tropoelastin monomers generated by VSMCs undergo extensive cross-linking to create mature elastin (Sandberg et al., 1981; Krettek et al., 2003), which is assembled into elastic fibers to form the elastin-contractile units around the VSMCs in the media. VSMCs are heterogenous and exhibit a high degree of plasticity (Alexander and Owens, 2012). In the media layer, VSMCs are the most abundant cell type and play key roles in maintaining structure and functions of the aorta (Lacolley et al., 2017). Upon circumferential stretching, the elastic fibers within the aortic wall store the energy created from the pumping left ventricle during systole and release the energy during diastole by returning to their initial dimension (Figure 2, upper panel). This is indispensable for maintaining a regular and efficient circulation in the peripheral tissues and organs during the cardiac cycle (Belz, 1995). Collagens closely associate with elastic fibers in the media (Dingemans et al., 2000) and contribute to aortic wall stiffness and strength (Wagenseil and Mecham, 2009; Wang et al., 2021). Collagen types I and III are enriched within the aortic wall; type I collagen localizes around the VSMCs, while type III collagen exists alongside the multiple layers of external elastic lamella in the media layer (Shekhonin et al., 1987). These series of cellular and ECM structural units in the media are the major determinants of the elastic properties of the aortic wall.

### Adventitia

The outermost layer of the aortic wall is the adventitia, composed of stem/progenitor cells, fibroblasts, pericytes, vasa vasorum (VV), lymphatic vessels, inflammatory cells (e.g., macrophages, dendritic cells, mast cells, T cells, and B cells), perivascular neurons, and adipocytes that are distributed in the collagen-rich connective tissue (Figure 1, left panel). ECM structures in the adventitia determine physical strengths of the aortic wall (Beenakker et al., 2012; Stenmark et al., 2013), which bears over half of the mechanical load to protect the aortic wall from overdistension. Importantly, adventitia is a regulatory center for vascular injury, and a pathological adventitia structure contributes to age-related vascular disease (Majesky et al., 2012; Stenmark et al., 2013; Tinajero and Gotlieb, 2020). Several types of stem/progenitor cells that reside adjacent to the media have the capacity to differentiate into VSMCs, ECs, chondrocytes, adipocytes, macrophages and myofibroblasts to maintain homeostasis of the aortic wall in response to physiological and pathological stimuli (Majesky et al., 2011; Majesky et al., 2012; Jolly et al., 2021). Fibroblasts that are absent in the intima and media are dispersed within the adventitia, where they serve to deposit collagen fibrils (Ross and Glomset, 1973; Kohn et al., 2015). VV is a specialized microvasculature that transports nutrients and oxygen to the adventitia and two-thirds of the media layer within the aortic wall. Adventitial lymphatic vessels modulate inflammation of the aortic wall by draining interstitial fluid and trafficking the immune cells through the aortic wall (Kutkut et al., 2015; Yeo et al., 2021). Perivascular innervation in the adventitia modulates functions of VSMCs and ECs to regulate vascular tone (Nava and Llorens, 2019). Perivascular adipose tissue (PVAT) also plays important roles in homeostasis of the aortic wall by releasing adipocytokines, chemokines and growth factors to determine inflammation status and stiffness of the aortic wall (Ozen et al., 2015; Nava and Llorens, 2019).

## Discussion

### Aging of Intima and Media

With aging, anti-aging factors (e.g., GDF11, IGF-1, klotho, oxytocin, nitric oxide (NO)) decrease, while pro-aging factors (e.g., β2-microglobulin, oxidized low-density lipoproteins) increase in the circulating blood (Ozen et al., 2015; Smith et al., 2015; Cannata et al., 2017; Yang et al., 2017; Sahu et al., 2018; Kang and Yang, 2020; Kiss et al., 2020; Rybtsova et al., 2020). ECs that cover the surface of blood vessels orchestrate arterial homeostasis by mediating vascular tone, coagulation, immune response, inflammation, metabolism and angiogenesis (Michiels, 2003; Busse and Fleming, 2006; Pober et al., 2009; Falkenberg et al., 2019; Kruger-Genge et al., 2019), and are constantly exposed to pro- and anti-aging factors in the blood. In concert with age-associated hemodynamic challenges (e.g., hypertension, decreased shear flow and increased turbulent flow resulting from dilated and tortuous aged aorta) (Lantz et al., 2015; Buford, 2016; Ha et al., 2018) and intrinsic cellular aging programs (Hohensinner et al., 2016; Morgan et al., 2018), degenerative imbalances in anti- and pro-aging factors directly or indirectly accelerate endothelial senescence in the aortic wall (Erusalimsky and Kurz, 2006). Senescent ECs mitotically halt and become dysfunctional (Donato et al., 2015; Bhayadia et al., 2016) (Figure 1 right panel), while exhibiting pro-inflammatory phenotypes (Wang et al., 2014a; Wang et al., 2014b; Pantsulaia et al., 2016) such as attenuated endothelial NO production, increased endothelin-1 (ET-1) release, production of inflammatory cytokines [e.g., IL-1, IL-6, IL-8, TNF-α, monocyte chemoattractant protein-1 (MCP-1)] (Maier et al., 1990; Pantsulaia et al., 2016), activation of surface adhesion receptors (e.g., ICAM-1, VCAM-1) and disruption of barrier function (Krouwer et al., 2012). Consequently, circulating immune cells are activated and adhere to the surface of dysfunctional ECs, which promotes their infiltration through the endothelium and into the subendothelial space (Trott et al., 2018) (Figure 1, right panel)*.* Activated macrophages also release pro-inflammatory cytokines such as TNF-α, IL-1b, IL-6 and INF-γ (Wang et al., 2007; Lesniewski et al., 2011a; Lesniewski et al., 2011b) and chronic, low-grade sterile inflammation ensues in the aortic wall (Wang et al., 2014a; Wang et al., 2014b; Duca et al., 2016) (Figure 1, right panel). In response to the age-associated proinflammatory microenvironment, degenerative ECM remodeling (e.g., degradation of BM, fragmentation of internal elastic laminae, excessive deposition and crosslinking of collagens) (Bruel and Oxlund, 1996; Tsamis et al., 2013; Steppan et al., 2019) leads to thickening and stiffening of the ECM network within the subendothelial space (Guyton et al., 1983; Lakatta and Levy, 2003) (Figure 1, right panel). As a result, ECs sense the substrate stiffness (Collins et al., 2014a), which in turn, further aggravates endothelial dysfunction (Fels et al., 2012). For example, stiffening of the subendothelial space due to aberrant ECM remodeling sensitizes ECs to oxidative stress, accelerates EC senescence (Urbano et al., 2019), and disrupts EC barrier function to further promote infiltration of activated immune cells (Huynh et al., 2011). Aberrant cell-ECM interactions also reduce NO bioavailability and impair endothelial-dependent vasodilation (Paar et al., 2014). Decreased NO bioavailability also stimulates activation of MMPs (Nascimento et al., 2019), which further degrades the matrix composition of the aortic wall, particularly within collagen and elastic fibers (Tronc et al., 2000; Upchurch et al., 2001; Zaragoza et al., 2002).

These changes in the intima layer contribute to aortic aging and pathogenesis of CVDs (inside-out theory: inner layer triggers remodeling of aortic wall) (Donato et al., 2015; Yin and Pickering, 2016). Physiological crosstalk between ECs, immune cells, ECM and VSMCs maintains homeostasis of the healthy aorta, while age-associated changes lead to deregulated crosstalk and aberrant aortic wall remodeling (Mendez-Barbero et al., 2021). Aged ECs and disorganized underlying ECM structures also synergistically stimulate migration of medial VSMCs into the intima (Li et al., 1999; Miller et al., 2007). These VSMCs aberrantly remodel native ECM structures in the intima (Schwartz et al., 1995) (Figure 1, right panel). Decreased expression of contractile proteins (Bochaton-Piallat et al., 1993; Bochaton-Piallat et al., 2001; Moon et al., 2003; Ferlosio et al., 2012) and increased expression of pro-inflammatory and migratory factors such as MMPs (Moon et al., 2003), MCP-1 (Spinetti et al., 2004) and PDGFR (Wang et al., 2012) in the VSMCs contribute to the intimal remodeling in the aging aorta (Li et al., 1999). These synthetic VSMCs further degrade elastic fibers, while deposit and crosslink collagens that are 100–1000 times stiffer than elastin between the fragmented elastic fibers in intima and media (Hays et al., 2018) (Figure 1, right panel). In the aging aorta, deregulated collagen deposition and crosslinking lead to fibrosis and thickening of the aortic wall (Movat et al., 1958; Spina and Garbin, 1976; Mccullagh et al., 1980; Toda et al., 1980; Andreotti et al., 1985; Shekhonin et al., 1987; Wang et al., 2006; Lyck Hansen et al., 2015). Aged ECs generate excess reactive oxygen species (ROS) through mitochondrial respiratory chain, NADPH oxidases and down regulation of antioxidant enzymes (Donato et al., 2015) to create chronic oxidative stress and subsequent low-grade inflammatory phenotype in the aortic wall (Ungvari et al., 2008), which leads to severe aortic pathologies such as atherosclerosis and abdominal aneurysm (AAA) (Ungvari et al., 2010; Peshkova et al., 2016; Golledge, 2019) in aged people. AAA is a 15th-leading cause of overall death in the United States and is an age-associated CVD (prevalence of men over 65 years is 4–8 percent) (Sakalihasan et al., 2018). The prevalence of aortic atherosclerosis also significantly increases along aging (Jaffer et al., 2002; Oyama et al., 2008; Chen et al., 2013) and causes serious CVDs such as coronary artery disease and stroke (Kronzon and Tunick, 2006; Di Tullio et al., 2008).

The heart beats over 100,000 times a day and each cardiac cycle induces pulsatile strain to the elastic fibers within the aortic wall. In the aged aorta, relatively quiescent elastic fibers in the media (Avolio et al., 1998) are mechanically fragmented and those fragmented elastic fibers are stabilized and stiffened by excessive deposition of collagens and their crosslinking in the pro-inflammatory environment (Duca et al., 2016). The fragmentation of the elastic fibers in the media with aging also enhances its proteolysis by specific elastases (i.e., elastolysis), which in turn generates short peptides known as elastin-derived peptides (Le Page et al., 2019). These peptides subsequently stimulate VSMC proliferation and differentiation into osteoblast-like cells (Simionescu et al., 2005) to accelerate inflammation and calcification of the aortic wall (Simionescu et al., 2005; Maurice et al., 2013; Dale et al., 2016). Importantly, exposure of the resident cells to the elastin-derived peptide further sustains chronic low-grade inflammation of the aortic wall (Le Page et al., 2019). Senescence of aged VSMCs (Monk and George, 2015) also accelerates aortic wall inflammation through the senescence-associated secretory phenotype (Chi et al., 2019) and osteoblast-like differentiation, which leads to calcium deposition in the media by upregulation of synthesis of alkaline phosphatase (ALP), bone morphogenetic protein (BMP), osteopontin (OPN), calpain-1, runt-related transcription factor (Runx-2) and collagens (Nakano-Kurimoto et al., 2009; Jiang et al., 2012; Fakhry et al., 2017). Calcium deposition around the elastic fibers is markedly increased in the aortic wall with aging (Lansing et al., 1950; Derlin et al., 2015), which further stiffens the medial layer and enhances aortic wall dysfunction (Laurent et al., 1996; Aronson, 2003).

Although arterial stiffening has historically been associated with changes in the native ECM structures, additional evidence suggests that stiffening of the resident cells [e.g., ECs and VSMCs (Gao et al., 2014; Nicholson et al., 2017; Morales-Quinones et al., 2020)] also contributes to overall stiffness of the aortic wall (Qiu et al., 2010; Sehgel et al., 2013; Sehgel et al., 2015; Leloup et al., 2019). The activity of the contractile filaments as well as the molecular signaling pathways that regulate actin polymerization and focal adhesion signaling in the VSMCs contribute to the age-associated vascular stiffening (Nicholson et al., 2018). Interactions between VSMCs and aberrant ECM structures also play key roles in these cellular events (Zhu et al., 2012; Ribeiro-Silva et al., 2021). Excessively deposited collagens progressively acquire crosslinks (Aronson, 2003) in the intima and media, altering their structural and functional properties. Thus, disorganized ECM structures, deregulated cell-ECM interactions, and increased cellular stiffness collectively contribute to the aortic wall stiffening phenotype.

### Aging of Adventitia

Adventitia not only provides structural support to the aortic wall, but also maintains its homeostasis (Tinajero and Gotlieb, 2020). Collagen fibers in the adventitia layer provide tensile strength and resilience to the cyclic deformation of the aortic wall caused by the beating heart (Thompson et al., 2002; Tsamis et al., 2013). As an axial load is placed on the vessel wall, the collagen fibers deform and straighten, exhibiting their high tensile strength. Adventitial fibroblasts synthesize and deposit collagen I and III, and these collagens increase with age and are also accompanied by stiffening of the aorta (Fleenor et al., 2010). While it is widely accepted that various adventitial cells (e.g., stem/progenitor cells, fibroblasts, arterial and lymphatic ECs, pericytes, immune cells, adipocytes, neuronal cells) and adventitial ECM molecules maintain homeostasis of the aortic wall, and deregulation of these adventitial components are proposed to contribute to pathological remodeling of the aortic wall such as atherosclerosis and AAA (outside-in theory: outer layer triggers remodeling of the aortic wall) (Stefanadis et al., 1995; Majesky et al., 2012; Majesky, 2015; Doderer et al., 2018; Tinajero and Gotlieb, 2020), the effects of aging on structure and functions of aortic adventitia have not been well-characterized compared to intima and media. A comprehensive analysis of cellular and non-cellular constituents of adventitia in young and aging aorta will be necessary to understand how they interact and crosstalk to maintain homeostasis of the aortic wall.

### Perspective

Life-long mechanical loads generated by the beating left ventricle and chronic low-grade inflammation stiffen the aortic wall with age, which accelerates EC dysfunction, and subsequent remodeling of the aortic wall. Importantly, normally aging aortic wall (Figure 1) is not pathological, but this is predisposed to CVDs such as atherosclerosis (e.g., formation of atheromatous plaque and foam cells) and AAA (e.g., medial necrosis, dissection and rupture). To prevent the aorta from progressing into these pathological conditions, it is critical to understand the structural and functional alterations of the aging aorta. These spiral cycles lead to impaired peripheral circulation, organ failure and cardiovascular events during aging (Thorin-Trescases and Thorin, 2016) (Figure 2). Several anti-aging remedies have been explored to prevent this over the decades. For example, anti-hypertensive medications, regular aerobic exercise habits or specific diets such as Mediterranean diet may slow down the vicious cycles and decrease the risk of CVDs (Vaitkevicius et al., 1993; Seals et al., 2008; Kanaki et al., 2013; Liu et al., 2013; Santos-Parker et al., 2014; Larocca et al., 2017; Upala et al., 2017). Although these anti-aging remedies may exert the beneficial effects by attenuating chronic low-grade inflammation (Chrysohoou et al., 2004; Lesniewski et al., 2011a; Lesniewski et al., 2011b; Woods et al., 2012), more comprehensive mechanisms of age-associated aortic wall remodeling need to be explored to develop more efficient anti-aortic aging strategies. Since stiffness of the aorta, which is determined by the structures of ECM and stiffness of the resident cells, can be measured in non-invasive ways using pulse-wave-velocity (PWV) (Cavalcante et al., 2011; Laurent et al., 2016; Salvi et al., 2019), more efficient anti-aortic aging strategies such as combination of medications, fitness program and diet instruction could be rigorously explored at the clinic level.

## Summary

Age-associated ECM alterations play crucial roles in the progression of aortic aging. Targeting altered ECM structures and cell-ECM interactions in the aortic wall using combinations of ECM and cytoskeleton modifiers can be a promising therapeutic strategy for aortic pathologies. Further functional characterization of the ECM alterations and cell-ECM interactions in the aged aorta will provide a new framework to develop more efficient therapies for aortic aging and CVDs.
